# The feminization of HPV: How science, politics, economics and gender norms shaped U.S. HPV vaccine implementation

**DOI:** 10.1016/j.pvr.2017.04.004

**Published:** 2017-04-23

**Authors:** Ellen M. Daley, Cheryl A. Vamos, Erika L. Thompson, Gregory D. Zimet, Zeev Rosberger, Laura Merrell, Nolan S. Kline

**Affiliations:** a Department of Community and Family Health, College of Public Health, University of South Florida, Tampa, FL, USA; b Section of Adolescent Medicine, Department of Pediatrics, Indiana University School of Medicine, Indianapolis, IN, USA; c Lady Davis Institute for Medical Research, Jewish General Hospital & Departments of Oncology, Psychiatry and Psychology, McGill University, Montreal, QC, Canada; d Department of Health Sciences, James Madison University, Harrisonburg, VA, USA; e Department of Anthropology, Rollins College, Winter Park, FL, USA

**Keywords:** HPV vaccination, Feminization, Critical review

## Abstract

Human papillomavirus (HPV) can cause a number of anogenital cancers (i.e., cervical, penile, anal, vaginal, vulvar) and genital warts. A decade ago, the HPV vaccine was approved, and has been shown to be a public health achievement that can reduce the morbidity and mortality for HPV-associated diseases. Yet, the mistaken over-identification of HPV as a female-specific disease has resulted in the *feminization of HPV* and HPV vaccines. In this critical review, we trace the evolution of the intersection of science, politics, economics and gender norms during the original HPV vaccine approval, marketing era, and implementation. Given the focus on cervical cancer screening, women were identified as bearing the burden of HPV infection and its related illnesses, and the group responsible for prevention. We also describe the consequences of the feminization of HPV, which has resulted primarily in reduced protection from HPV-related illnesses for males. We propose a multilevel approach to normalizing HPV vaccines as an important aspect of overall health for both genders. This process must engage multiple stakeholders, including providers, parents, patients, professional organizations, public health agencies, policymakers, researchers, and community-based organizations.

## 1 Introduction

Vaccinations are one of the top public health achievements of the early twenty-first century, and the HPV vaccine has the potential to prevent morbidity and mortality from cervical and other HPV-related cancer and diseases [1]. The initial HPV vaccine was approved a decade ago for use in the United States, and although it has been justifiably lauded as a scientific triumph for disease prevention, it also has been a source of controversy for various reasons since its 2006 approval. The vaccine's development and implementation trajectory has been specifically focused on females due its initial testing and marketing to prevent cervical cancer [2], resulting in direct and indirect gender biases and corresponding inequities for HPV-related diseases. Consequently, HPV and its associated interventions have become *feminized*. Feminization occurs when an issue's social construction concentrates on females [3]. The term feminization has historical roots in the context of poverty during the 1970's and later with the HIV epidemic. In the case of its 1970's association with poverty, complex social and economic issues such as unequal pay for equal work, and increasing divorce rates that led to more women heading single-parent households introduced the new phenomenon of gender-related poverty. Feminization was associated with HIV when African American women were identified in the early 2000's as having the highest rates of HIV acquisition in the U.S., a new phenomenon that changed the “face” of HIV. With both phenomena, the issue was characterized as female-focused and layered with perceptions of vulnerability, power imbalances, and gender disparities [4], [5], [6]. Additionally, a type of reproductive technology that has also been feminized is contraception. Specifically, contraception is more costly for women (financially and physically), and women are the focus of procreative responsibility, thus bearing the burden of the contraception responsibility [7]. This feminization of contraception also generates a strong cultural and social perception of contraception as feminine, thus excluding males [8].

Feminization has the potential to negatively impact public awareness and approaches to address social and health issues across multiple stakeholder levels (e.g., government, organizations) [4], [5], [6]. In this review, we trace the intersection of science, politics, economics, and gender norms during the original development, approval and marketing phase of the HPV vaccine, in which HPV was characterized as solely impacting women. In doing so, we extend a longstanding tradition of critical and feminist scholarship that considers how health sciences can be used in ways to “discipline” and “regulate” women's bodies by including HPV in such conversations. [9], [10] Accordingly, we review how the interplay of science and sexism has contributed to the *feminization of HPV* and fits within an existing cultural narrative of HPV being a woman's problem. We conclude by evaluating the impact of feminization of HPV by examining the HPV vaccine's current utilization and disparities in the United States, skewed economic discussions for HPV vaccine policy, and insufficient protection for males.

## 2 Cervical cancer, HPV and the HPV vaccine

As early as five centuries ago, the relationship between cervical cancer and sexual transmission was postulated [11], [12], [13]. Since the 1970's, scientific inquiries focused on the connection between papillomavirus infection and cervical cancer, which demonstrated the presence of HPV DNA in cervical cancer and genital warts [14], [15], [16]. In 1983 Harald zur Hausen identified HPV-subtype 16 DNA in precursor cervical cancer tumors and two years later established that HPV DNA was present in cervical cancer cells [17]. Following this breakthrough, lab-based and epidemiological studies evolved, which elucidated the connection between HPV and cervical cancer (see zur Hausen [18] and International Agency for Research on Cancer [19] for detailed information). This science laid the foundation for future HPV prevention approaches.

By the year 2000, cervical cancer was the second most common cancer among women worldwide and approximately half of all women who had cervical cancer died annually [20]. The global burden from cervical cancer was far greater in developing nations than in developed nations that had implemented highly effective screening programs, which resulted in lower rates of cancer deaths [21]. This global burden propelled the continued focus on HPV's connection to cervical cancer, and the quest to identify a vaccine. Critical to the development of the HPV vaccines, virus-like particles technology allowed for the mechanism to mimic HPV capsid proteins, thus producing neutralizing antibodies without actually including live HPV DNA in the vaccine [22], [23], [24]. Successive clinical trials assessed the safety and efficacy of vaccines protecting against HPV-types 16 and 18 [25], [26], [27], and HPV-types 6, 11, 16, and 18 [28], [29], [30]. Cervical cancer typically develops over many years, and identifying an early marker that acted as a “surrogate” endpoint was critical of early vaccine trials. The clinical endpoints of these trials were either persistent infection with HPV–targeted types or cervical/genital disease among women [31].

After years of global vaccine research and development, within the United States the Food and Drug Administration (FDA) reviewed the primary evidence of a quadrivalent HPV vaccine (4vHPV; meaning that four types of HPV - 6, 11, 16 and 18 - were targeted) [32]. This discussion focused on the efficacy of the vaccine among females 16-to-26 years of age for cervical, vaginal, and vulvar cancers and their precursors, and whether immunogenicity data supported the extension to females 9-to-15 years of age. Given that the sole focus on the vaccine clinical trial data was on females, the resulting FDA approval of the vaccine was female specific [32]. Table 1 illustrates this complex timeline for U.S. HPV vaccine approval and recommendations for 2006–2016. In 2006, the Advisory Committee on Immunization Practices (ACIP) issued their first recommendation for the vaccine for females 11-to-12, and approval for the vaccine to be administered in females ages of 9 through the “catch-up” ages of 13-to-26 years [33]. Following the 4vHPV approval, in 2009, a bivalent vaccine (2vHPV; types 16 and 18) also was approved by the FDA, but for females 10-to-25 years of age. Subsequent recommendations that same year by ACIP expanded the FDA indication for routine vaccination of females 11-to-12 years, with the same 4vHPV approval protocol from ages 9-through-26 years [34]. The success of these vaccines, particularly 4vHPV, is demonstrated by a 64% decrease in HPV types 6, 11, 16, and 18 prevalence among 14-to-19 year old females 6 years after the vaccine's introduction [35].

**Table 1:** Timeline of FDA approvals and ACIP recommendations for the HPV vaccine in the United States.

**Year**	**Month**	**Agency**	**Vaccine**	**Recommendation/Approval**
2006	June	FDA	**4vHPV**	Approved vaccine for use in females 9–26 years of age
	June	ACIP	**4vHPV**	Recommended routine vaccine for females 11–12 years; catch-up 13–26 years; can be started at age 9

2009	October	FDA	**2vHPV**	Approved vaccine for use in females 10–25 years of age
	October	ACIP	**2vHPV**	Recommended vaccination for females 11–12 years; catch-up 13–26 years; can be started at age 9
	October	FDA	**4vHPV**	Approved vaccine for use males 9–26 years of age
	October	ACIP	**4vHPV**	Recommended vaccination may be given to males age 9–26 years – did not recommend routine vaccination

2011	October	ACIP	**4vHPV**	Recommended routine vaccination for males 11–12 years; catch-up 13–21 years and catch-up 22–26 years for men who have sex with men (MSM) or are immunocompromised; can be started at age 9
				
2014	December	FDA	**9vHPV**	Approved use in females 9–26 years of age
			**9vHPV**	Approved use in males 9–15 years of age

2015	February	ACIP	**9vHPV**	Recommended routine vaccination for females 11–12 years; catch-up 13–26 years; can be started at age 9
			**9vHPV**	Recommended routine vaccination for males 11–12 years; catch-up 13–21 years and catch-up 22–26 years for MSM and men who are immunocompromised; can be started at age 9
	December	FDA	**9vHPV**	Approved use in males 16–26 years of age

2016	October	FDA	**9vHPV**	Approved use of a two-dose option for males and females 9–14 years
	December	ACIP	**9vHPV**	Recommended two-dose option for males and females 9–14 years

## 3 Implications for male HPV vaccination

In addition to identifying HPV as a cause for cervical cancer, other studies investigated HPV's connection to anogenital cancers, including penile and anal cancers [36], [37]. The examination of the natural history of HPV in men occurred a decade after similar studies among women [38]. Consequently, the lag in the epidemiological evidence for HPV in males hindered corresponding vaccine recommendations for males. Three years after initial approval of the first HPV vaccine, ACIP issued a statement in 2009 that males *may* be vaccinated with 4vHPV to prevent genital warts – a “permissive” rather than “routine” approval for use in males [39]. In 2011, with evidence that 4vHPV prevented anal pre-cancers, ACIP updated the recommendation for males indicating that males 11-to-12 years should be *routinely* vaccinated, males 13-to-21 should be routinely vaccinated as a catch-up group, and males 22-to-26 who are in high-risk populations, such as men who have sex with men (MSM), should be vaccinated [40]. The extension of the age recommendation to high-risk subgroups was the result of cost-effectiveness data indicating that MSM are at higher risk of anal cancers and genital warts [41], and would receive additional benefit from vaccination at later age ranges compared to heterosexual males. The delay in these recommendations for males was attributed to limited data regarding HPV's role in anal cancer and genital warts, and concerns about the cost-effectiveness of vaccinating males [34], [39], [40].

## 4 The evolution of the HPV vaccine to the present

**Fig. 1:**
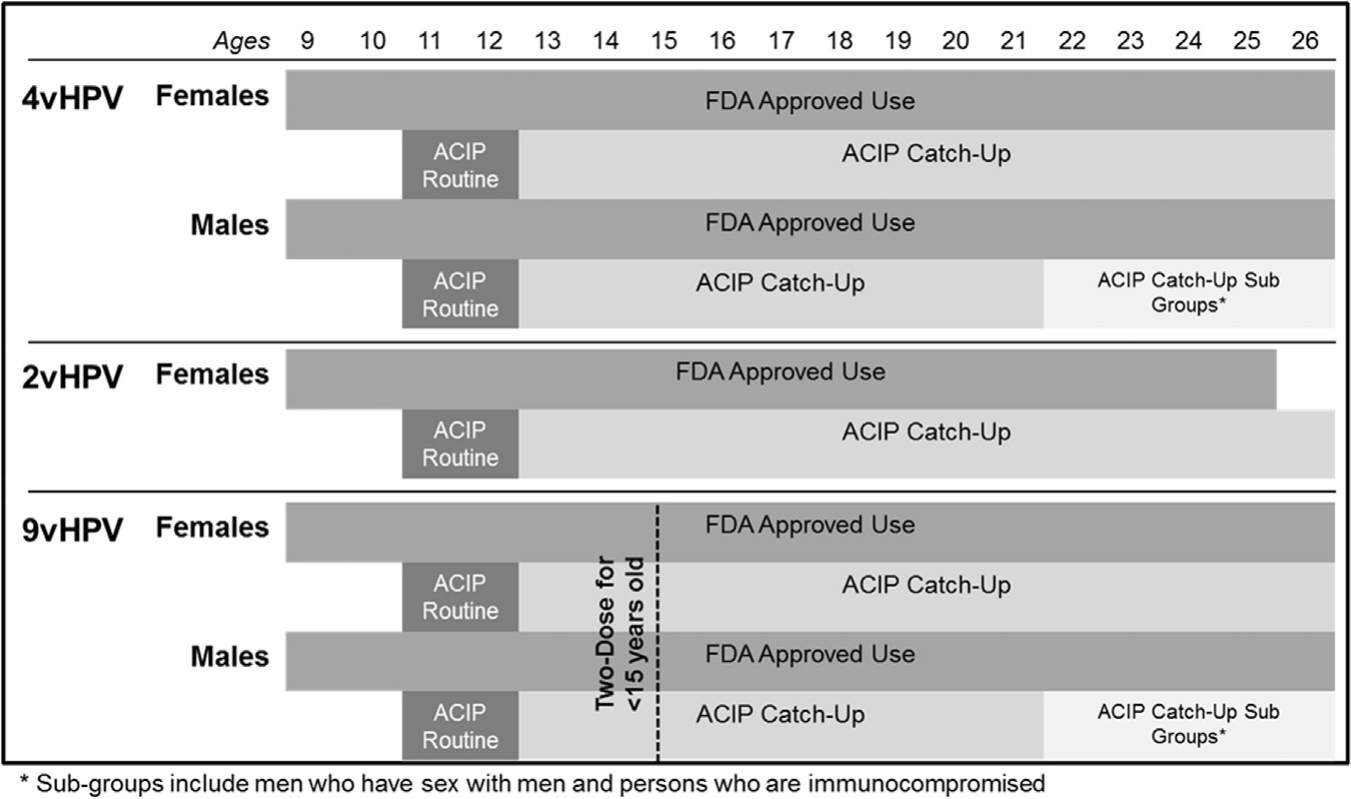
Current U.S. approvals and recommendations for HPV vaccination by sex, age, and vaccine type.

Evolving research during the decade since the vaccine was first approved has resulted in changes both in the vaccine itself, and in the dosing schedule. However, policy differences between the two federal agencies (the FDA and ACIP) responsible for the approval of the vaccine have resulted in confusing recommendations by age, sex, and vaccine type (Fig. 1). In December 2014, the FDA approved a 9-valent HPV vaccine (9vHPV) for females 9-to-26 years of age and in males from 9-to-15 years of age, but with ACIP subsequently issuing recommendations in March 2015 that mirrored 4vHPV [42], [43]. 9vHPV protects against nine HPV types [6, 11, 16, 18, 31, 33, 45, 52 and 58], seven of which are oncogenic, and 9vHPV is 97% effective for preventing cervical, vaginal, and vulvar cancers caused by the identified HPV types, and 78% effective for preventing anal cancer [42], [44]. Approximately one year later in December 2015, the FDA modified approval for use of 9vHPV for males to ages 16-to-26 years for the prevention of anal cancer and genital warts [45]. Most recently, in October 2016 the FDA approved and in December 2016 ACIP recommended a two-dose option for 9vHPV for males and females ages 9 through 14 years, Individuals must be vaccinated before age 15, and follow a 0 and 6–12 month vaccine delivery schedule [46], [47]. These inconsistencies across the HPV vaccine approval timeline have contributed to impediments to achieving greater coverage in vaccination uptake and reducing the burden of disease (Fig. 1). Furthermore, the continued gender-based difference in recommended guidelines serves to imply that HPV disproportionately affects one gender over another, when in fact both groups are burdened by HPV-related cancers and genital warts.

## 5 Politics and policy of the HPV vaccine in the United States

While a number of other countries utilize school-based approaches for nationwide vaccine programs [48], the U.S. relies on vaccine dissemination primarily through healthcare providers [31]. Even with national ACIP recommendations provided for HPV vaccination, implementation policy varies state-by-state. Previous and current state efforts have attempted school-entry mandates [49], yet many of these legislative efforts have been unsuccessful (e.g., Kentucky, New York, New Mexico, Texas). Initially, the majority of the legislative bills focused on educating, providing coverage, or requiring the HPV vaccine for females only; more recent legislative efforts are transitioning to be more inclusive of males as well [49]. Only the state of Virginia and the District of Columbia have been successful in passing and implementing legislation requiring HPV immunization HPV for school-entry [50]. Virginia's law, however, includes very liberal opt-out language (i.e., *philosophical exemption*) that makes enforcement impossible, and for both Virginia and the District of Columbia, only girls are included in the legislation, perpetuating the focus on protection of girls [50]. It was not until 2014 that the legislation in the District of Columbia was amended to also include males in this vaccine policy [50]. An alternative strategy for HPV vaccination mandates is through health departments [51]. In 2015, Rhode Island became the first state to successfully implement a gender-neutral HPV vaccine school-entry requirement, which was institutionalized through the state health department rather than through legislative means [49].

Due to the separation of state and federal lawmaking in the United States, individual states can determine and implement state-specific legislation regarding HPV vaccination requirements and mandates. According to the National Conference of State Legislatures, 42 states and territories have introduced legislation related to HPV vaccine requirements, funding, or education. Yet some states are more successful than others in the passage of these initiatives [49]. While policy related to the HPV vaccine may be the most critical strategy for improving uptake of the HPV vaccine, it is clear that state-level and community-level politics play an essential role in the state and local legislators. Thus, political will is required to promote HPV vaccine legislation in all states and territories.

## 6 The feminization of HPV: consequences for females

From a scientific standpoint, the creation of the HPV vaccine was straightforward and an almost perfect case-study of the development of a highly efficacious and safe vaccine. A more critical investigation, however, reveals how the vaccine developed in tandem with pervasive gender biases. HPV vaccine research logically proceeded in women because of the known cytological site of cervical infection and an easily identified “surrogate” endpoint. There was not an identified cancer in males that had the prevalence – or the clinical endpoints – that rivaled cervical cancer, and although genital warts affect males and females, no males were involved in the initial vaccine trials and the primary impetus for vaccine development was cancer prevention. The initial vaccine was approved for adolescent females [33], based upon the available data and the societal context in which the vaccine was discovered.

Although typically characterized as a woman's disease, HPV is sexually transmitted (skin to skin, genital to skin, and oral to genital), implying contact with another person [52]. It is estimated that nearly 85% of women and 91% of men with at least one sexual partner from the opposite sex will contract HPV infection during their lifetime [53]. Moreover, males are more likely to have HPV in their lifetime and be the recipients of HPV in heterosexual transmission studies [54]. These data underscore that HPV is not a gender-specific infection.

Feminization of HPV reinforces the long-held belief that women are responsible for reproductive healthcare in heterosexual partnerships [8], [55]. Traditionally, secondary prevention efforts for HPV and cervical cancer are solely the responsibility of women as HPV screening is not available for men [56], further reinforcing this belief. Concomitantly, it assigns the stigma and blame toward women as both the host *and* transmitting agent of HPV [57]. Thus, females are burdened with screening and treatment of HPV-related diseases, while males both fail to obtain the primary prevention they need, and accurately perceive their risk for infection and disease.

Heuristic beliefs that the HPV vaccine is associated with early sexual behavior among adolescents persist, yet scientific evidence repeatedly demonstrates that this is not the case [58], [59], [60]. Social and cultural discomfort with female adolescent sexuality is not new. It stems from a prolonged uneasiness with female sexuality and long held sexual double standards for males and females. Similar reproductive health topics, such as adolescent pregnancy, are intractably tied to young females, who are seen as threatening to society, as well as in need of protection [61]. The effects of these sexist undertones may be hindering widespread vaccination efforts. For example, Taylor et al. [62] suggest parents may delay vaccination past the target range of 11-to-12 years for females until later in adolescence since sexuality is not a key concern at this time period.

In addition to the scientific research trajectory, the initial marketing of the HPV vaccine to females in the U.S. further feminized HPV in the public mind. As noted above, the 4vHPV vaccine, marketed by Merck Pharmaceuticals, was approved by in 2006, a full 3 years before Glaxo Smith-Kline's 2vHPV vaccine, Cervarix, was approved. The GSK version of the vaccine has never had a strong presence in the U.S. In expectation of the 2006 licensure of the 4vHPV vaccine, Merck & Co., Inc. released two unbranded awareness campaigns in June of that year, *Tell Someone* and *Make the Connection*, targeted at adult women. These campaigns show women, including a mother and daughter dyad, expressing their shock at the connection between HPV infection and cervical cancer and their determination to ‘tell’ everyone in their lives they care about, including their sister and mother [63]. After the licensure of Gardasil in October of 2006, Merck released their “One Less” national print, television, and online advertising campaign targeting mothers and young women on November 13, 2006. The preliminary 4vHPV marketing (“One Less” campaign [64]) understandably framed the vaccine from the cervical cancer prevention standpoint and voiced female empowerment as a reason for vaccination [65]. The declaration of vaccinated girls being “one less” exemplifies this rhetoric. An unfortunate and unintended consequence of this campaign was a skewed view that HPV impacted only females [66]. Additionally, this initial marketing campaign failed to directly address the sexual transmission of HPV and the implications for the prevention of genital warts [67], [68]. Given that the goal of the marketing campaign was to increase vaccine uptake, it is likely that Merck attempted to avoid these discussions among the American public who often stigmatize, politicize, and misrepresent sexual health issues [68], [69]. In 2008, Merck launched their “I Chose” campaign aimed at catch-up vaccination aged women. This campaign continues the themes seen in the “One Less” campaign, with women being empowered to protect themselves from “two types of HPV that cause cervical cancer, and the two types that cause other HPV diseases.” However, the ‘other HPV diseases’ are never identified. Although changes in HPV vaccine guidelines led to an expansion of marketing for the vaccine that included males, undertones of HPV as a “woman's disease” continued to permeate popular media. Moreover, newly developed gender-neutral, direct-to-consumer advertisements for the vaccine were generally restricted to women's magazines that targeted mothers with adolescents [66].

## 7 The feminization of HPV: consequences for males

The feminization of HPV has had numerous consequences, disadvantaging males from receiving a beneficial vaccine and continuing a sexist rhetoric of HPV primarily impacting females. From a public health standpoint, if vaccine uptake rates in females were adequate, a treatise on the feminization of HPV would be essentially unnecessary because of achieving herd immunity. Unfortunately, we are nowhere near that threshold. Data on 2014 4vHPV uptake showed that 60% of females and 42% of males 13-to-17 years had received at least one dose and 40% of females and 22% of males completed the vaccine series [70]. Similar trends are observed more distinctly among the catch-up vaccine groups; among women 18-to-26 years in 2012 the uptake rate was 34% [71], while among males the uptake rate in 2011–2012 was only 5.5% [72]. The inequity in the uptake rates among age groups and genders is indication of the complicated and inconsistent HPV guidelines and corresponding timelines. It further illustrates the misunderstanding that vaccination against HPV is equally important for both males and females. Indeed, reports on HPV vaccination in the U.S. typically have focused on low HPV rates among females, not males [35], [73]. This has unfortunately contributed to the misalignment of the vaccine with females only.

Because the initial approach in the United States focused on achieving high vaccination rates among females, cost-effectiveness models have been inadvertently skewed, basing the value of vaccination only upon one gender. This has mistakenly framed the question as, “*is it cost-effective to vaccinate both males and females compared to females only*” rather than asking “*is it cost-effective to vaccinate both genders compared to not vaccinating anyone*?” Additionally, cost-benefit models tend to underestimate the benefit of male vaccination, as they include assumptions regarding high female vaccine coverage [74]. Assessing the cost-effectiveness of the HPV vaccine through gender-based herd immunity is unlike any other immunization policy [75].

There is an additional inherent limitation to the herd immunity thesis as it is based on heteronormative worldviews. The initial logic of the HPV vaccine and herd immunity ignores MSM, and therefore does not achieve sufficient protection for all males, especially among a sub-group at greater risk for HPV-related outcomes. Clinical trial data among MSM who have been vaccinated with 4vHPV demonstrate a reduced risk of anal cancer [76], an important outcome for this population that has higher rates of this cancer [77]. The combination of a complicated cost-effectiveness approach and heteronormativity has resulted in stratified and confusing HPV vaccine guidelines for males, based on sexuality. This can consequently make it more difficult to MSM to receive the HPV vaccine during “catch-up” years. Some males may have reservations about disclosing sexual orientation or same-sex sexual behavior to a healthcare provider; similarly, some healthcare providers may not be aware of, or ask about, a patient's sexual behavior, resulting in missed opportunities for HPV vaccination [78], [79].

The connection of HPV to cervical cancers has overshadowed the importance of preventing other HPV-related cancers, including oropharyngeal cancers, which affect both males and females. Oropharyngeal cancer is the second most common HPV-related cancer in the U.S., and it is projected that by 2020, HPV-related oropharyngeal cancers will exceed HPV-related cervical cancers [80]. In 2009, new cases of oropharyngeal cancers accounted for 37.3% of all HPV-associated cancers, whereas cervical cancers represented 32.7% [81]. It is estimated that 9vHPV could potentially prevent between 6000 and 8000 oropharyngeal cancer cases a year among males – nearly three times the number of cases among females [82], [83]. Yet, there are no straightforward pre-clinical endpoints for oropharyngeal cancers, nor are there any approved screening techniques similar to cervical cancer [84], making it difficult to conduct rigorous shorter-term studies demonstrating that HPV vaccination prevents pre-oropharyngeal cancers. This problem has limited the indications for the HPV vaccine to the prevention of anogenital diseases. Assuming HPV vaccination does prevent oropharyngeal cancers, then an unfortunate consequence of the vaccine being over-identified with females is insufficient protection for males from these serious cancers.

As a result of the delay in male HPV vaccine approval, studies have indicated that females have a greater awareness and knowledge of the HPV vaccine compared to males [85], [86]. This is most likely attributable to media and health messages that have targeted females for a longer period of time. Females and parents of females receive far more consistent and strong healthcare provider HPV vaccine recommendations compared to males [87], [88], [89], an issue of particular concern in the U.S. because of the importance of provider recommendation for vaccine dissemination as opposed to school-based programs [90], [91], [92]. Finally, males in the older age of the vaccine catch-up category (22-to-26 years) may face additional obstacles to receiving the vaccine, not only due to heteronormative policies, but due in part to “aging out” of pediatric practices, since males typically do not follow guidelines for annual exams similar to female gynecological exams [93].

## 8 Reversing the feminization of the HPV vaccine

The *feminization of HPV* involves a complicated story intersected by science, politics, economics and gender norms. Historical events leading up to the HPV vaccine were driven by both scientific and economic priorities, necessitating the initial licensure for females. Given these priorities, and the reliance upon scientific evidence to drive policy, retrospective decisions to approve the vaccine for both males and females from 9-to-26 years would have been untenable. Nonetheless, the failure to create consistent guidance between males and females at the earliest opportunity underscores the risk of fragmenting science and policy.

It is encouraging that science and public health policy have begun to bridge the gap between scholarly evidence and societal norms to recognize that HPV vaccination is important for *all* young people. This is demonstrated by Healthy People 2020 objectives that call for increase in HPV vaccine 3-dose coverage among 13-to-15 year old females *and* males to 80% [94]. Unfortunately, rates of vaccination in the U.S. are dismal compared to the public health targets. Thus, implementation of these national objectives will be challenging. We believe that a *national movement* is required to implement state-by-state legislation and policy for school-based entry for HPV vaccination that is gender neutral. This will require coordination from multiple stakeholders: healthcare providers, parents, young adults, state-level policymakers, public health agencies, and professional organizations. Normalization of HPV vaccination is needed for this prevention strategy, despite associated controversy. Moreover, if we fail to face the issue of feminization of HPV directly, we will continue to perpetuate these consequences.

Thus, in order to improve HPV vaccination, we suggest a multilevel approach in normalizing HPV vaccines as an important aspect of overall health for all individuals. This approach includes policy strategies, provider education and communication tools, targeted patient messages, and coordinated action among providers and scholars. Borrowing from the social ecological model of health, we assert that a gender-neutral strategy for HPV vaccination must involve key agents at every level of influence to normalize HPV as gender-neutral [95]. Additionally, this strategy must consider the longstanding gender beliefs that contribute to the feminization of HPV. Disentangling adolescent female sexuality and protection of women only from these messages are required for a gender-neutral approach.

At a policy level, we advocate normalizing HPV vaccination through school entry requirements with strict exemption guidelines, identical to those for other adolescent vaccines. In making the HPV vaccine a requirement for school entry, the vaccine can instead be viewed as one of the vaccines necessary for school-age children. While requiring HPV vaccination for school entry would potentially normalize the vaccine and increase uptake, initial resistance to normalizing HPV vaccination through schools may persist. Additionally, we must consider the implementation of previous HPV vaccine mandates that have been less than successful, and learn from these efforts. For example, Virginia enacted a school-entry mandate in 2008 for HPV vaccination among girls, but this mandate had a *philosophical exemption* that has largely contributed to the lack of improvement in vaccination rates [96]. In contrast, Rhode Island's HPV vaccine mandate does not include this language, and may prove to be more successful, as it does not include lenient exemption policies [96]. We argue for strict exemption guidelines that are limited to religious or medical exemptions only.

In addition to normalizing HPV vaccination through policy, normalization must occur through primary care physicians (e.g., pediatricians, family practice, and OB/GYNs). Existing evidence suggests these physicians may be hesitant to recommend HPV vaccines [97], but providers play an important role in determining whether patients complete a vaccine series. Accordingly, we suggest strong statements from providers’ professional organizations and accompanying communication tools that give providers an effective and persuasive means through which to recommend HPV vaccination to parents. Moreover, professional organizations must encourage all clinical staff to discuss and promote the HPV vaccine since staff members can play supportive roles in reinforcing positive health messages. Provider-focused messaging is especially important in the current time period given the advent of 9vHPV, which allows for providers to contextualize new HPV messages as a form of emerging evidence to reframe existing narratives about HPV. The introduction of 9vHPV and eventual discontinued use of 4vHPV provides an ideal time for providers to discuss this topic with parents and explain the latest vaccine as a way to protect *all* people from HPV-related disease.

Similarly, the emergence of 9vHPV provides an opportunity for public health agencies such as the CDC to implement messages targeting both unvaccinated individuals and parents with vaccine-eligible children. Rather than reproducing the gendered messages of 4vHPV, 9vHPV messages must include gender-neutral information that challenges existing views of HPV. Such messages can normalize HPV vaccination by emphasizing its importance for males and females in the same regard as other vaccines. These messages can simultaneously defeminize HPV and emphasize the importance of *all* vaccines for protecting against preventable causes of death and disease. Agencies should design these messages in consultation with academic researchers, providers, and community organization groups to ensure their relevance in advancing gender-neutral HPV ideals.

## 9 Conclusion

HPV has a long history of feminization that has been perpetuated by the intersection of science, politics, economics and gender norms. Not only has the feminization of HPV supported historic rhetoric of gender inequality, which adversely impacts females, but has also directly impacted males by resulting in lower-than-expected HPV vaccination rates. In our suggestions for normalizing HPV vaccination and making it gender neutral, we assert a need for multilevel approaches targeting providers, patients, parents, professional organizations, public health agencies, policymakers, researchers, and community-based organizations. This approach can combat the consequences of feminization and potentially increase the likelihood of changing existing assumptions about HPV.
